# Porcine cis-acting lnc-CAST positively regulates CXCL8 expression through histone H3K27ac

**DOI:** 10.1186/s13567-024-01296-9

**Published:** 2024-05-07

**Authors:** Junxin Gao, Haidong Yu, Yu Pan, Xinrong Wang, He Zhang, Yunfei Xu, Wenjie Ma, Wenli Zhang, Lizhi Fu, Yue Wang

**Affiliations:** 1 College of Veterinary Medicine, Southwest University, Chongqing, 400715 China; 2 Harbin Veterinary Research Institute, Chinese Academy of Agricultural Sciences, Harbin, 150069 China; 3 Chongqing Academy of Animal Science, Chongqing, 408599 China; 4 National Center of Technology Innovation for Pigs, Chongqing, 402460 China

**Keywords:** CXCL8, lnc-CAST, PRRSV, chemokine regulation

## Abstract

The chemokine CXCL8, also known as the neutrophil chemotactic factor, plays a crucial role in mediating inflammatory responses and managing cellular immune reactions during viral infections. Porcine reproductive and respiratory syndrome virus (PRRSV) primarily infects pulmonary alveolar macrophages (PAMs), leading to acute pulmonary infections. In this study, we explored a novel long non-coding RNA (lncRNA), termed lnc-CAST, situated within the *Cxcl8* gene locus. This lncRNA was found to be highly expressed in porcine macrophages. We observed that both lnc-CAST and CXCL8 were significantly upregulated in PAMs following PRRSV infection, and after treatments with lipopolysaccharide (LPS) or lipoteichoic acid (LTA). Furthermore, we noticed a concurrent upregulation of lnc-CAST and CXCL8 expression in lungs of PRRSV-infected pigs. We then determined that lnc-CAST positively influenced CXCL8 expression in PAMs. Overexpression of lnc-CAST led to an increase in CXCL8 production, which in turn enhanced the migration of epithelial cells and the recruitment of neutrophils. Conversely, inhibiting lnc-CAST expression resulted in reduced CXCL8 production in PAMs, leading to decreased migration levels of epithelial cells and neutrophils. From a mechanistic perspective, we found that lnc-CAST, localized in the nucleus, facilitated the enrichment of histone H3K27ac in CXCL8 promoter region, thereby stimulating CXCL8 transcription in a cis-regulatory manner. In conclusion, our study underscores the pivotal critical role of lnc-CAST in regulating CXCL8 production, offering valuable insights into chemokine regulation and lung damage during PRRSV infection.

**Supplementary Information:**

The online version contains supplementary material available at 10.1186/s13567-024-01296-9.

## Introduction

Swift production of chemokines is a critical response to virus infection, playing a pivotal role in the recruitment of neutrophils, controlling virus replication and spread, and ultimately influencing the outcomes of the infection. However, excessive levels of chemokines and the consequent exaggerated infiltration of neutrophils during viral infection have been strongly linked with inflammatory tissue damage and adverse outcome. This has been observed in infections such as Severe Acute Respiratory Syndrome Coronavirus 2 (SARS-CoV2), influenza virus, and porcine reproductive and respiratory syndrome virus (PRRSV) [1–4]. Currently, strategies targeting chemokines or inflammatory cytokines have emerged as promising treatments for inflammatory diseases [5–7]. For example, the administration of monoclonal antibodies targeting α-chemokine interleukin-8 (also known as CXCL8 or IL-8) has demonstrated a significant reduction in clinical disease activity in cases of palmoplantar pustulosis [8].

CXCL8, also recognized as neutrophil chemotactic factor, is an inflammatory chemokine produced by a variety of cell types, including macrophages, epithelial cells, fibroblasts, and hepatocytes [9]. As the primary cells to recognize antigens, macrophages are likely the first to release CXCL8 at sites of infection or injury [10]. CXCL8 plays a vital role in inducing chemotaxis in target cells, primarily neutrophils, as well as other cell types such as endothelial cells, macrophages, and keratinocytes. This effectively guides these cells to migrate to infection sites [11]. Additionally, CXCL8 has been identified as an important mediator of responses to numerous bacterial and respiratory virus infections, including PRRSV infections [3, 12, 13]. In the context of PRRSV infection, porcine pulmonary alveolar macrophages (PAMs) serve as the primary target cells for viral replication in vivo. PRRSV infection is associated with severe lung lesions, characterized by the destruction of lung structure, extensive hemorrhage, and interstitial pneumonia. Elevated levels of proinflammatory chemokines, including CXCL8, have been implicated in the infiltration of immune cells into the lungs [14–17]. Hence, the precise regulation of CXCL8 production is essential for maintaining a controlled immune reaction.

An increasing body of evidence suggests that long non-coding RNAs (lncRNAs) play various roles in biological processes, including the modulation of transcription, the architecture of the nucleus, and the regulation of proteins or RNA molecules [18, 19]. Some lncRNAs serve as structural molecules, recruiting histone-modifying enzymes either locally (*cis*) or distantly (*trans*) [20]. Others, transcribed from enhancer regions, facilitate the transcription of protein-coding genes [21, 22]. Furthermore, a significant number of lncRNAs have induced and implicated in the pathogenesis of human diseases [23]. However, our understanding of the precise molecular mechanisms underlying the involvement of lncRNA in infections remains limited, particularly in terms of their functions of lncRNAs in the host’s antiviral response. Therefore, the aim of this study was to investigate a specific porcine lncRNA and explore its potential functions in inflammation induced by viral infection.

## Materials and methods

### Cells, animals, and viruses

Primary and immortalized porcine alveolar macrophages (PAMs) were cultured in RPMI Medium 1640 basic (Life Technologies, Carlsbad, California, USA), supplied with 10% fetal bovine serum (FBS, Hyclone, Logan, Utah, USA). Marc-145 (a monkey kidney cell line), PK-15 (a porcine kidney cell line), PT-K75 (pig intranasal mucosal fibroblasts), and HEK-293T (human embryonic kidney) were cultured in Dulbecco’s minimum essential medium (Life Technologies, USA), also supplied with 8% FBS (Hyclone, USA), 100 U/mL penicillin, and 100 µg/mL streptomycin. The SPF pigs used in the experiments were sourced from Harbin Veterinary Research Institute of Chinese Academy of Agricultural Sciences. The animal experiments conducted were approved by the Animal Care and Use Committee of Harbin Veterinary Research Institute of Chinese Academy of Agricultural Sciences (Approval ID: 200720-01). All experiments were performed in accordance with the regulations and guidelines established by this committee. The PRRSV North American-like strain HuN4 (GenBank accession number EF635006) was grown and titrated in Marc-145 cells, as previously described, and was stored at −80 °C.

### 5′ and 3′ RACE

The Rapid Amplification of cDNA Ends (RACE) PCR was conducted using the SMARTer PCR cDNA Synthesis Kit (TaKaRa, Kusatsu, Shiga, Japan). Specific and nested primers were utilized for both 5′ and 3′ RACE. Following the 5′ and 3′ RACE, full-length lnc-CAST was amplified using the PrimeStar DNA polymerase (TaKaRa, Japan). The amplification conditions were as follows: 98 °C for 2 min, followed by 30 cycles of 98 °C for 5 s, 60 °C for 5 s, and 72 °C for 3 min, and a final extension at 72 °C for 5 min. The PCR products were subsequently cloned into the T vector (TaKaRa, Japan). The specific and nested primers used in this process are listed in Additional file 1.

### Identification and analysis of full-length lnc-CAST

The entire transcriptome library was prepared and sequenced, and the raw and processed RNA-seq data has been deposited into the NCBI database under accession number: Bioproject PRJNA658105. This includes Biosamples SAMN15857517 to SAMN15857534. For the prediction of lncRNAs, we retained only those transcripts that were longer than 200 nucleotides, had more than one exon, and had an optimal expression threshold of FPKM > 0.5 in at least one sample.

### Total RNA extraction and quantitative PCR

Total RNA was extracted from both cells and tissues using TRIzol (Invitrogen, Carlsbad, California, USA) and was subsequently reverse transcribed into cDNA using PrimeScript RT reagent Kit (TaKaRa, Japan). This process was conducted according to the manufacturer’s instructions. The Real-Time Quantitative PCR (RT-qPCR) was performed on a QuantStudio 5 system (Applied Biosystems, USA), utilizing SYBR premix Ex Taq (TaKaRa, Japan). Fold changes were calculated using the cycle threshold method (ΔΔCT) [24]. The primers used in this process are listed in Additional file 2.

### ELISA

To measure the production of CXCL8 in tissues, we collected samples from pig lungs. These samples were then ground and processed to create a tissue suspension for detection. To measure the production of CXCL8 by PAMs, cells were transfected with lentiviral-CAST/siRNA for 48 h. Subsequently, cell supernatants were collected. The quantification of CXCL8 was performed using an IL-8 ELISA Kit (Invitrogen, USA), in strict accordance with the manufacturer’s instructions.

### siRNA transfection

The process of siRNA transfection was performed as per our previous description [24]. The target cells were transfected with 100 nM siRNA, specifically targeting lnc-CAST (5′-GGUGGAAGAAACCAACAAATT-3′), or the corresponding negative control (5′-UUCUCCGAACGUGUCACGUTT-3′). This was done over a period of 24 h using Lipofectamine™ RNAiMAX (Invitrogen, USA) according to the manufacturer’s instructions. The siRNAs were synthesized by GenePharma (Shanghai, China). Post-transfection, the cells were analyzed using RT-qPCR and/or Western blot to evaluate the efficiency of silencing and to monitor the expression of CXCL8.

### Plasmids

To overexpress lnc-CAST, we amplified its full length from PAMs cDNA using the primers listed in Additional file 3. This was then cloned into the pLVX-IRES-ZsGreen1 plasmid. The plasmid, carrying lnc-CAST, was co-transfected with pSPAX2 and pVSV-G into HEK-293T cells at a final concentration of 3 μg/mL (The ratio of pLVX-IRES-ZsGreen1:pSPAX2:pVSV-G was 3:2:1). This was done using the X-tremeGENE HP DNA transfection reagent (Roche, Basel, Switzerland), as per the manufacturer’s instructions. The vector pLVX-IRES-ZsGreen1, along with pspax2 and VSVG, served as the negative control. After 48 h of co-transfection, the cells were inoculated with the supernatant of HEK-293T cells. Following an incubation period of 1 h, the cell monolayers were washed and further incubated in fresh culture media for an additional 48 h or at indicated time points. Finally, the cells and supernatants were collected separately for analysis via RT-qPCR and Western blot.

For the Dual-luciferase reporter assays, the CXCL8 promoter was recombined into pGL3-Basic vector. The pRL-TK plasmid and pGL3-Basic vector, integral to this process, were purchased from Promega, USA. The primers used are detailed in Additional file 3. All plasmids underwent verification through sequencing of the pertinent regions. These plasmids were then extracted using the Endotoxin-Free Plasmid DNA Miniprep Kit (Tiangen, Beijing, China), strictly adhering to the manufacturer’s instructions.

### Western blotting

The Western blotting analysis was performed as previously described with a slight modification [25]. PAMs were inoculated with either lentiviral-CAST or a negative control. At 48 h post-infection, the cells were lysed using Pierce IP lysis buffer (Thermo Scientific, Rockford, IL, USA). The lysates were then separated by SDS-PAGE under reducing conditions and transferred onto a PVDF membrane. Following a blocking step, the membrane was incubated with the appropriate primary and secondary antibodies. The membranes were subsequently scanned and analyzed using an Odyssey instrument (Li-Cor Biosciences, Lincoln, Nebraska, USA). The Anti-β-actin antibody was purchased from Invitrogen, USA, while the anti-CXCL-8 antibody and HRP-conjugated goat anti-rabbit IgG were from Abcam.

### Transwell assay

PK-T75, IPEC or PK15 cells were detached from culture plates, washed twice in DMEM (Life Technologies, USA), and resuspended at a density of 5 × 10^5^/mL. A cell migration assay was conducted using 24-well plates/case (Corning Inc, Corning, NY, USA). We added 200 μL of the cell suspension to the upper chamber of an insert with an 8.0 μm pore size, and a 6.5 mm diameter, in triplicate. To the lower chamber of each well, we added 500 μL of either DMEM, supernatants from differently treated PAMs, or 20% FBS DMEM. The cells were then incubated at 37 °C for 24 h to allow migration. Following incubation, the plate was removed, and the cells were fixed with 4% methanol (Invitrogen, USA) in PBS. The membranes were stained with 0.1% crystal violet in PBS for 20 min, then thoroughly destained with water. Non-migratory cells on the upper surface of the membrane were removed by wiping with a tip, and the membranes were mounted on a glass microscope slide. Cells were counted in 5 random fields per membrane at a magnification of 200×.

For the transmigration assays, porcine neutrophils were isolated from peripheral blood, washed, and resuspended (1 × 10^6^ cells/mL) in fresh DMEM. These cells were then added to the top of a monolayer using an insert with an 8.0 μm pore size and 6.5 mm diameter. We added 500 μL of either DMEM, supernatant of PAMs, or DMEM carrying 20% FBS to the lower chamber of each well. The cells were then incubated at 37 °C for 2 h to allow migration. Following this, we analyzed the cells using a flow cytometric assay.

### Wound-healing assay

For the wound-healing assay, 2 × 10^5^ PK-T75, IPEC or PK15 cells were seeded in 6-well plate, and cultured overnight. Once the cells reached approximately 80% confluence, we created scratch using a sterile plastic tip. The cells were then incubated with the supernatant of PAMs transfected with either lentiviral-CAST or a negative control. Images of the plates were captured using a microscope at 0 h and 24 h post-scratch. The data were subsequently analyzed using ImageJ.

### Digoxigenin-labeled RNA

Digoxigenin (DIG)-labeled RNA probes were produced using a method adapted from the introductions of DIG Northern Starter Kit (Roche, Switzerland). Briefly, DNA templates were prepared from total RNA using RT-qPCR with oligo(dT) Primer. The PCR was then run with specially designed primers, including the sequence of the T7 RNA polymerase promoter. The sequence of the specific probe for Northern blot analysis of lnc-CAST was as follows:

GGTAAGTTTTTTAAAAATTTTGATTATTTATCTTGTGACATACAGTAAAAGTTAGCTTATAAATTTCCAGCTGCTGCTGCAAATTTTCTCTTTTAAGTATCTGCCTCTTTGATGAAAAACAAACAAACAAAGAAATTAGCAGCAACAGTGAGCTTCTTGTACTTGTAATTGGGAGGTAATGCATCCAGAAAGTATGGGCAAGCGTCTTGCCCTGGATCAGTAGTATGTCTCCCTCTCAATCTCAGAAGTGTTTAGATGACAACTTATTTAACCTCTCTATTTATAGTTGAAAATATAGGGTATTTCTAACACATAAATATCTAAGACCCTATTAATATCTATATTAGATATATTTTCAAGAGCGTACATTCCTCCTTATTTAAGTACCGTTCGTTTTTAACTTTTTTAAAAATTTCATTTATTTTTA. The RNA probes were labeled and designed by DNA template in a vitro transcription reaction with DIG-11-UTP using a labeling mixture and an optimized Transcription Buffer. The yield of DIG-labeled RNA was determined using a direct detection method in comparison to the control RNA.

### Northern blot

RNA Northern blots were run using a method adapted from the introductions of DIG Northern Starter Kit (Roche, Switzerland). In brief, 100 ng of RNA sample per lane was mixed with 20 μL loading buffer and incubated at 65 °C for 10 min. The RNA samples/loading buffer mix was then run on 2% formaldehyde gels in RNase-free gel boxes at 3–4 V/cm for at least 2 h until the RNAs were well separated. The 2% formaldehyde gels were then blotted by capillary transfer with 20 × SSC overnight or at least for 6 h. After that, the wet membrane was UV-crosslinked without prior washing. DIG-labeled RNA probes were boiled for 5 min, rapidly cooled in ice to denature, and added to the membrane at 68 °C with gentle agitation and incubation for 6 h. After hybridization and stringency washes, the membrane was briefly rinsed in washing buffer for 5 min, incubated for 30 min in blocking solution, and then washed 2 × 15 min in washing buffer. The membrane was equilibrated for 5 min in detection buffer, and 1 mL CDP-Star was added. The membrane was immediately exposed to an imaging device for 5–20 min or to X-ray for 5–20 min at 15–25 °C.

### RNA fluorescence in situ hybridization (FISH) and immunofluorescence microscopy

To detect lnc-CAST, PAMs from SPF pigs were rinsed briefly in PBS and then fixed in 4% formaldehyde in PBS (PH 7.4) for 30 min. PAMs were permeabilized in PBS containing 0.1–0.2% Triton X-100 (Invitrogen, USA) and washed in PBS for three times. Hybridization was carried out using DIG-labeled (Roche, Switzerland) lnc-CAST RNA probes at 37 °C overnight. After hybridization, cells were incubated with anti-digoxin-rhodamine Fab fragments (Roche, Switzerland) for 2 h and subjected to confocal microscopic imaging. For colocalization studies, cells were incubated again for 2 h in 2% BSA, subjected to immunofluorescence staining of H3K27ac/β-actin using anti-H3K27ac/anti-β-actin mAb (Abcam, Waltham, Boston, USA), and subjected to DAPI staining. PAMs were then observed with a Zeiss (LSM880, Oberkochen, Germany) confocal laser scanning microscope.

### Nuclear and cytoplasmic extraction of lncRNA

PAM cells isolated from lungs of SPF pigs were subject to nuclear and cytoplasmic extraction of RNA using the PARIS™ Kit protein and RNA Isolation System (Invitrogen, USA), following the manufacturer’s instructions. The nuclear and cytoplasmic fractions of RNA of PAMs were then subjected to RT-qPCR analyses of lncRNA and electrophoresis analysis of PCR products. The primers are listed in Additional files 1 and 2.

### Dual-luciferase reporter assay

pGL3-basic luciferase reporter vectors and pRL-TK-Renilla-luciferase vectors were co-transfected using Lipo2000 regent (Invitrogen, USA) on 5 × 10^5^ HEK-293T cells. At 12 h after transfection, HEK-293T cells were treated by lentivirus-mediated overexpress of lnc-CAST or negative control for 12 h. At 12 h after lnc-CAST treatment, HEK-293T cells were lysed in 200 µL lysis buffer, and the firefly luciferase and Renilla activities were determined with a luminometer by the Dual Luciferase Report Assay System (Promega, Madison, Wisconsin, USA). The primers are listed in Additional file 3.

### Chromatin immunoprecipitation followed by quantitative PCR (ChIP)

ChIP analysis was performed on 2 × 10^6^ cells using the EZ-Magna ChIP A/G Kit (Millipore, Burlington, Massachusetts, USA) following the manufacturer’s instructions. The H3K27ac antibody was from Abcam, USA. The positive control (anti-RNA Polymerase II) and the IgG negative control were from Millipore, USA. RT-qPCR amplification was carried out in a QuantStudio 5 system (Applied Biosystems, Foster City, California, USA) using SYBR premix Ex Taq (TaKaRa, Japan). As a control, input DNA was purified from chromatin before IP was used. The primers are listed in Additional file 2.

### Statistical analysis

All statistical analyses were carried out using Prism 8.0.1 and Excel. We performed at least three independent reproducible results for most experiments. Differences between the experimental and control groups were tested by using two-way ANOVA with Bonferroni’s post-test. Data are presented as the mean ± standard deviations (SD) from three or more independent experiments. A *p*-value of < 0.05 was considered statistically significant.

## Results

### Identification of lnc-CAST in porcine macrophages

In our previous study, we identified a group of lncRNAs that were associated with immune responses during PRRSV infection. These lncRNAs exhibited regulatory effects on their potential target genes in PAMs [26]. However, the precise function of lncRNAs in regulating inflammatory responses in PAMs remains elusive. Notably, pigs infected with PRRSV displayed an influx of immune cell populations, including neutrophils, mast cells, and macrophages, responding to the viral infection in the lungs. This was accompanied by elevated levels of the chemokine CXCL8 [16]. This observation prompted us to investigate whether certain lncRNAs are closely associated with the increased production of CXCL8. Through a comprehensive analysis of differentially expressed lncRNAs available in the NCBI database (accession number Bioproject PRJNA658105), we identified lnc_022171. This lncRNA was upregulated in PRRSV-infected PAMs and located in close proximity to the *Cxcl8* gene locus, suggesting that this particular lncRNA may function as a local effector. Utilizing RACE assay, we successfully obtained a novel transcript in *Cxcl8* gene locus, which consisted of 1515 nucleotides (Additional files 4A and 4B). This entire transcript had a 5′ end derived from intron 1 of the *Cxcl8* gene, shared the same sequence with introns 2, 3 and 4 of *Cxcl8*, and contained polyadenylated downstream from a consensus poly(A) in 3′ end. This is dramatically different from the pre-mRNA of the *Cxcl8* gene (Additional file 4C). Moreover, analyses employing the Coding Potential Calculator and Open Reading Frame Finder revealed the non-coding nature of this transcript (Additional file 4D). Consequently, we designated this transcript as the CXCL8 Active-Specific Transcript (referred to as “lnc-CAST”).

**Figure 1:**
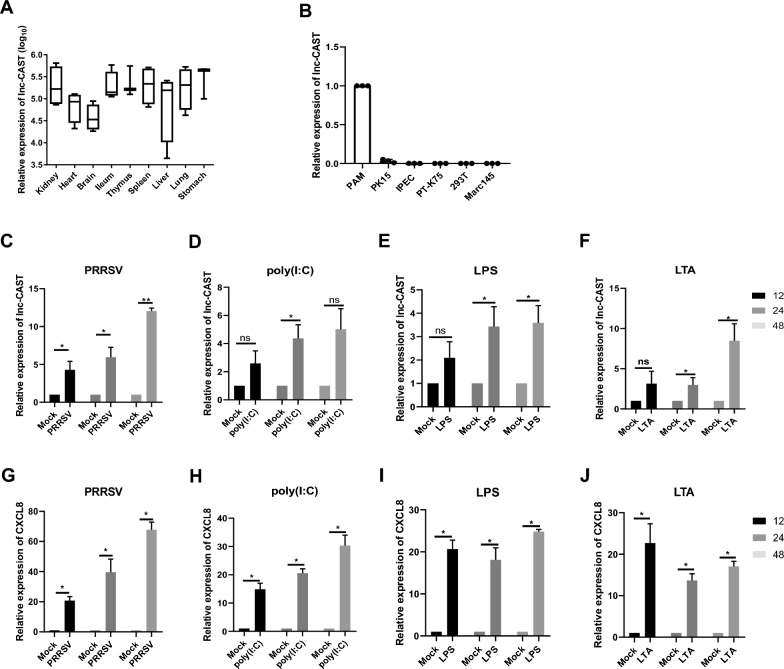
**Identification of lnc-CAST in porcine macrophages.** **A** Define the molecular distribution of lnc-CAST in vivo. Relative expression levels of lnc-CAST in porcine tissues in terms of liver, heart, ileum, thymus, spleen, stomach, brain, kidney and lung analyzed by RT-qPCR from SPF pigs. **B** Expression levels of lnc-CAST from six different cells (293T, PK15, IPEC, PAM, Marc-145, PT-k75). Expression levels of selected genes were measured, and data represent three independent experiments. Statistical significance (ANOVA test): *P < 0.05. **C**–**F** Upregulation of lnc-CAST in PAM cells following PRRSV infection, poly(I:C), LPS, and LTA stimulation were measured using RT-qPCR. **G**–**J** Upregulation of CXCL8 transcript in PAM cells following PRRSV infection, poly (I:C), LPS, and LTA stimulation were detected using RT-qPCR. Data were analyzed by two-sided *t* tests. Values that are significantly different are indicated by a bar and asterisks as follows: *P < 0.05; **P < 0.01.

Given that the majority of lncRNAs display tissue-specific expression patterns [27], we investigated the distribution of lnc-CAST across different tissues. Our analysis using RT-qPCR revealed that lnc-CAST was constitutively expressed at high levels in several porcine tissues, including the kidney, ileum, spleen, lung, and stomach (Figure 1A). Intrigued by the widespread expression of lnc-CAST, we extended our investigation to its expression in various cell lines. As illustrated in Figure 1B, lnc-CAST showed robust expression exclusively in PAMs, but not in structural cells. This includes PK-15, IPEC, PT-K75, 293T, and Marc145.

To gain a deeper understanding of the characteristics of lnc-CAST, we further evaluated its expression in primary PAMs following PRRSV infection. Notably, RT-qPCR analyses revealed a consistent increase in lnc-CAST expression at 24 h-post-infection (hpi) compared to the control (Figure 1C). Subsequently, we examined the expression levels of lnc-CAST in response to transfection with poly(I:C), a synthetic double-stranded RNA mimic. As depicted in Figure 1D, lnc-CAST expression progressively increased following poly(I:C) transfection. To assess the involvement of lnc-CAST in the inflammatory response, we incubated PAMs with either LPS or LTA. Interestingly, we observed an over-time increase in the expression level of lnc-CAST in cells treated with either LPS or LTA compared to the negative control (Figures 1E, F). Simultaneously, we investigated the expression levels of CXCL8 in PAMs upon treatment with PRRSV, poly(I:C), LPS or LTA. As shown in Figures 1G–J, CXCL8 expression exhibited a dramatic upregulation in response to all four treatments, displaying a significant correlation with the expression of lnc-CAST (R^2^ = 0.97). Collectively, these findings underscore the pivotal role of lnc-CAST in orchestrating immune response triggered by inflammatory stimulation, including PRRSV infection.

### Lnc-CAST expression is increased in PRRSV infection-induced lung inflammation

**Figure 2:**
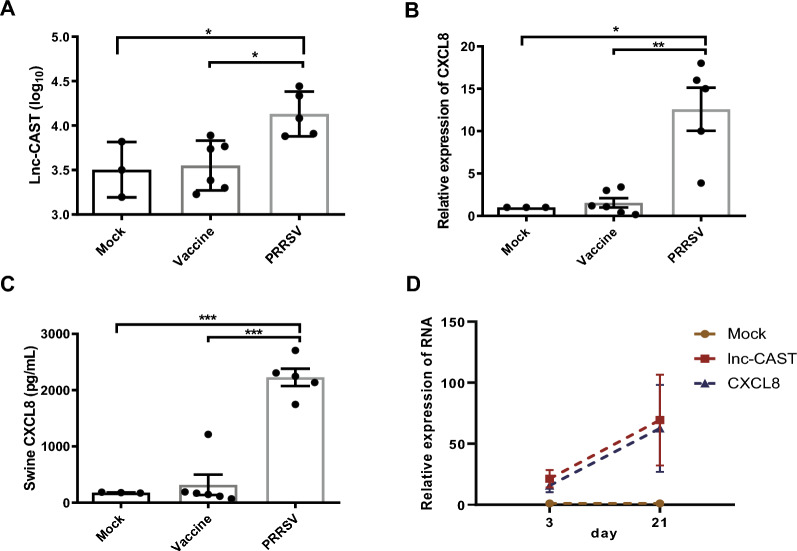
**Define the lnc-CAST or CXCL8 expression in vivo for treatment groups, including mock, vaccine strain HuN4-F112 and high-pathogenic PRRSV strain HuN4 inoculated pigs.** **A** RT‐qPCR was used to detect expression levels of lnc-CAST in lung tissues in HuN4-inoculated pigs, compared with mock and HuN4-F112 pigs, demonstrating that lnc-CAST was upregulated in PRRSV-infected group for 21 days. **B**, **C** mRNA and protein levels of CXCL8 in PRRSV-infected lung tissues was upregulated for 21 days. **D** Levels of expression of both lncRNA lnc-CAST and CXCL8 in lung tissue were analyzed by RT‐qPCR, and which showed the similarly upregulated trend on 3 days and 21 days. Statistical significance (ANOVA test): *P < 0.05; **P < 0.01.

To validate our initial findings, we delved deeper into the biological implications of lnc-CAST within the framework of lung inflammation induced by PRRSV infection. We gathered lung tissue samples from pigs subjected to various conditions, including mock inoculation, immunization with the PRRSV vaccine strain HuN4-F112, and infection with the highly pathogenic PRRSV strain HuN4. This was carried out over a span of 21 days, as previously described [28]. The RT-qPCR results revealed a significant upsurge in the expression levels of lnc-CAST in pigs inoculated with HuN4, compared to both the mock controls and the HuN4-F112 vaccinated pigs (Figure 2A). In a similar vein, HuN4 infection led to a notable increase in the transcriptional levels of CXCL8, when contrasted with the control group or the HuN4-F112 vaccinated pigs (Figure 2B). This was further corroborated by ELISA results, which confirmed a significant rise in the production of soluble CXCL8 protein in lung tissues following HuN4 infection (Figure 2C). Moreover, we observed that the expression levels of both lnc-CAST and CXCL8 were significantly elevated in pigs infected with HuN4 on day 21, as opposed to day 3 (Figure 2D). Collectively, these observations suggest that lnc-CAST is upregulated during PRRSV infection, which in turn triggers a feedback mechanism that induces the production of CXCL8 in vivo.

### Lnc-CAST positively regulates CXCL8 expression in PAMs

**Figure 3:**
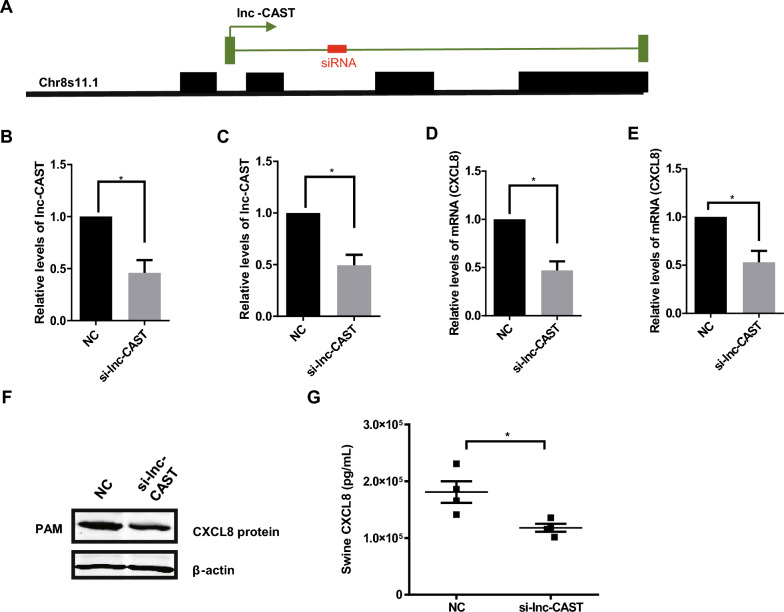
**Lnc-CAST regulates CXCL8 expression in PAMs.** SiRNA treatment decreased the expression of lnc-CAST transcript and significantly attenuated expression and release of CXCL8 protein in PAM cells. **A** Schematic illustration of siRNA for lnc-CAST. **B**, **C** Impact of siRNA knockdown of lnc-CAST in primary and immortalized PAM cells. **D**, **E** Knockdown of lnc-CAST in primary and immortalized PAM cells attenuated CXCL8 expression as assessed using RT-qPCR. **F** Knockdown of lnc-CAST altered protein expression of CXCL8 in primary PAM cells by Western blot. **G** Treatment of PAM cells with the siRNA to lnc-CAST, but not the nonspecific siRNA controls, attenuated the release of CXCL8. Two-sided t tests were used to compare RNA or protein levels between samples. Values that are significantly different are indicated by a bar and asterisks as follows: *P < 0.05.

To assess the regulatory influence of lnc-CAST on CXCL8 expression, we engineered a siRNA that targets the intron 3 region of lnc-CAST, referred to as “si-lnc-CAST” (Figure 3A). RT-qPCR results indicated that the transfection of si-lnc-CAST into primary PAMs led to a significant reduction in lnc-CAST expression (Figure 3B). Analogous results were observed in immortalized PAMs (Figure 3C). Crucially, the decline in lnc-CAST expression was associated with a significant decrease in CXCL8 mRNA levels in both primary and immortalized PAMs (Figures 3D, E). To validate the impact of si-lnc-CAST on CXCL8 expression, we gathered cell lysates and cell supernatants from the siRNA-transfected PAMs. Western blot analyses unveiled a decrease in cell-associated CXCL8 protein expression post si-lnc-CAST transfection (Figure 3F). Concurrently, ELISA results highlighted a significant reduction in soluble CXCL8 protein levels in si-lnc-CAST-transfected PAMs (Figure 3G). These findings collectively suggest that downregulation of lnc-CAST inhibits the production and release of CXCL8 protein.

**Figure 4:**
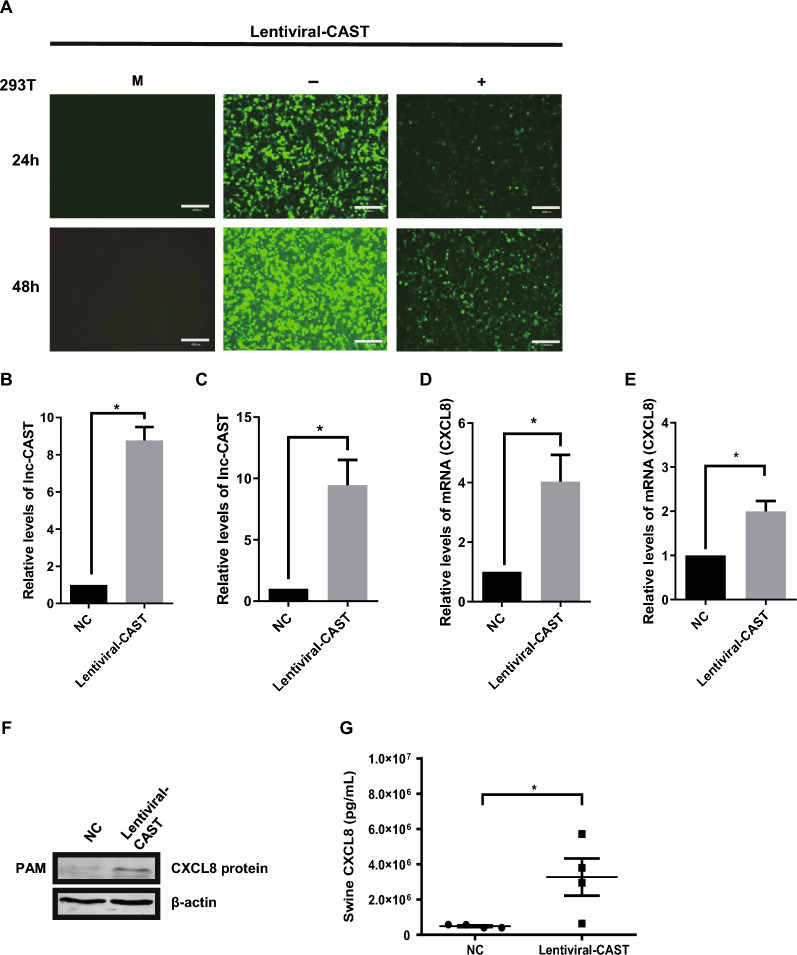
**Overexpressed lnc-CAST induced upregulation of CXCL8 transcript and significantly promoted expression and release of CXCL8 protein in PAM cells.** **A** Lentiviral vector carrying lnc-CAST has been successfully constructed and maintains high expression in HEK-293T cells. M, untreated control; −, empty lentiviral vector control; +, lentiviral vector carrying CAST. **B**, **C** Overexpression of lnc-CAST in primary and immortalized PAM cells. **D**, **E** Upregulation of lnc-CAST in primary and immortalized PAM cells promoted CXCL8 expression as assessed using RT-qPCR. **F** Upregulation of lnc-CAST enhanced protein expression of CXCL8 in primary PAM cells by Western blot. **G** Treatment of PAM cells with lentiviral-CAST upregulated the release of CXCL8 in the supernatant. Expression level of CXCL8 was validated by using ELISA (*t* test, *P < 0.05).

To further confirm the role of lnc-CAST in regulating CXCL8 expression, we constructed a lentiviral vector embedded with lnc-CAST (hereafter referred to as “lentiviral-CAST”) to facilitate the overexpression of lnc-CAST in PAMs [29]. The efficiency of this transduction was determined by GFP expression (Figure 4A). RT-qPCR analyses validated the successful overexpression in both primary and immortalized PAMs treated with lentiviral-CAST (Figures 4B, C). As shown in Figures 4D, E, the transcription levels of CXCL8 were significantly increased in cells overexpressing lnc-CAST. Moreover, an elevation was observed in both cell-associated and extracellular CXCL8 protein levels in primary and immortalized PAMs overexpressing lnc-CAST (Figures 4F, G). Altogether, these results indicate that lnc-CAST acts as a *cis*-acting regulator for the production of CXCL8 in PAMs, implying a potential involvement of lnc-CAST in inflammation-related innate immune responses.

### Lnc-CAST affects the migration capacity of target cells

**Figure 5:**
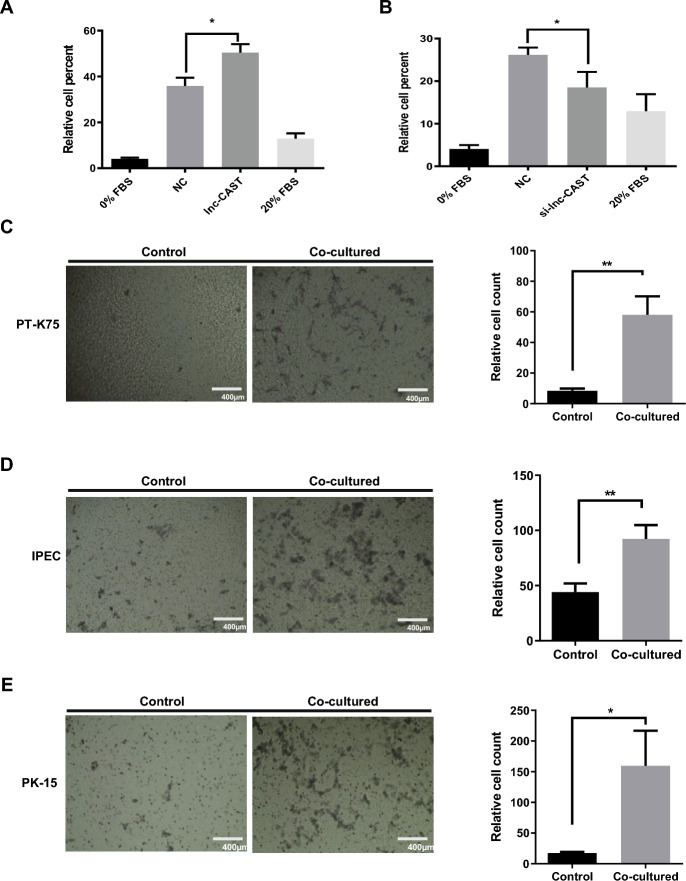
**Lnc-CAST affects the migration capacity of target cells.** **A** Supernatants from lnc-CAST overexpressed PAMs resulted in the increase of neutrophil migration in contrast to the control, examined by flow cytometry. **B** Percentage of neutrophil migration was significantly suppressed in supernatants of si-lnc-CAST transfected PAMs using flow cytometry assay. Data represent three independent experiments. Statistical significance (ANOVA test): *P < 0.05. The capacity of cell migration was analyzed by Transwell assay for **C** PT-K75 cells, **D** IPEC cells, and **E** PK-15 cells after co-culturing with increased lnc-CAST supernatant. Data were analyzed by ImageJ (*t* test, *P < 0.05; **P < 0.01). Values that are significantly different are indicated by a bar and asterisks as follows: *P < 0.05; **P < 0.01.

Given that CXCL8 is recognized as a neutrophil chemotactic factor, we processed to investigate the potential impact of lnc-CAST on neutrophil migration. This was accomplished by utilizing cell supernatants derived from PAMs that either overexpressed lnc-CAST or carried a control vector. As illustrated in Figure 5A, we observed an increase in neutrophil migration in the presence of supernatants from PAMs overexpressing lnc-CAST, compared to the control. Conversely, a significant reduction in the percentage of neutrophil migration was noted when using supernatants obtained from cells transfected with si-lnc-CAST (Figure 5B). These findings suggest that lnc-CAST possesses the ability to augment the production of the chemoattractant CXCL8, which is essential for neutrophil migration.

In addition to inducing chemotaxis in neutrophils, CXCL8 is also known to stimulate the migration of other cell types to sites of inflammation [30]. Consequently, we explored the influence of lnc-CAST on the migration of other cell types, including IPEC, PT-K75, and PK15 cells. An equal number of these cells were seeded in triplicate in the upper chamber of Corning’s Transwell permeable supports, following the procedure outlined in the Materials and methods section. Supernatants from PAMs overexpressing lnc-CAST, serving as a chemoattractant, were added to the bottom chamber, and the cells were subsequently incubated under standard conditions for 24 h. Post-incubation, the migratory cells were stained and quantified. As demonstrated in Figure 5C, there was an approximate fivefold increase in the migration of PT-K75 cells compared to the control. Similarly, IPEC cells showed a twofold increase in migration (Figure 5D), and a comparable trend was observed in PK-15 cells (Figure 5E). These findings lend support to the hypothesis that lnc-CAST-mediated CXCL8 production contributes to enhanced cell migration.

### Lnc-CAST regulates wound repair capacity of structural cells in vitro

**Figure 6:**
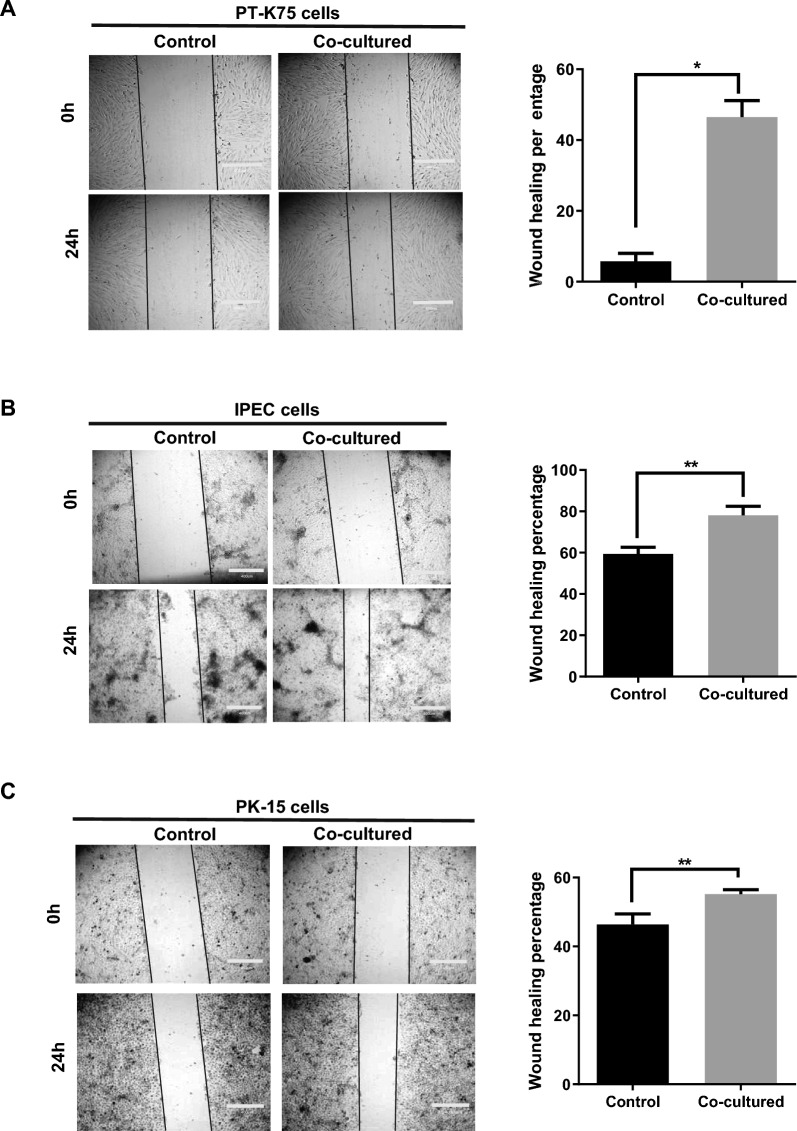
**Lnc-CAST regulates wound repair capacity of structural cells in vitro.** The wound repair capacity of cell migration in **A** PT-K75 cells, **B** IPEC cells, and **C** PK-15 cells was analyzed by hound healing assay after co-culturing with increased lnc-CAST supernatant for 24 h. Data were analyzed by ImageJ (*t* test, *P < 0.05; **P < 0.01). Values that are significantly different are indicated by a bar and asterisks as follows: *P < 0.05; **P < 0.01.

To further explore the regulatory role of lnc-CAST, we conducted in vitro wound-healing assays. The impact of lnc-CAST on IPEC, PT-K75, and PK-15 cells was assessed in an “end-time” assay, carried out as previously described with minor modifications [31]. In brief, the three cell lines were cultured until they reached confluence, at which point single-path wounds were created. Given that wound repair is facilitated by migration, we monitored and evaluated wound closure at 24 h post-injury. As shown in Figure 6A, the most robust wound repair was observed in intranasal mucosal fibroblast PT-K75 cells, which were co-cultured with supernatants from PAMs overexpressing lnc-CAST, in comparison to the control. Notably, weaker yet significant responses were also detected in porcine intestinal epithelial cell IPEC treated with the supernatants (Figure 6B). Similarly, incubation of porcine kidney cell PK-15 with the supernatants led to an enhancement in wound repair (Figure 6C). These results suggest that the secretion of soluble CXCL8, mediated by lnc-CAST, triggers significant migration of target cells into single-path wounds, thereby promoting enhanced wound repair.

### Lnc-CAST promotes CXCL8 expression as a potential enhancer

**Figure 7:**
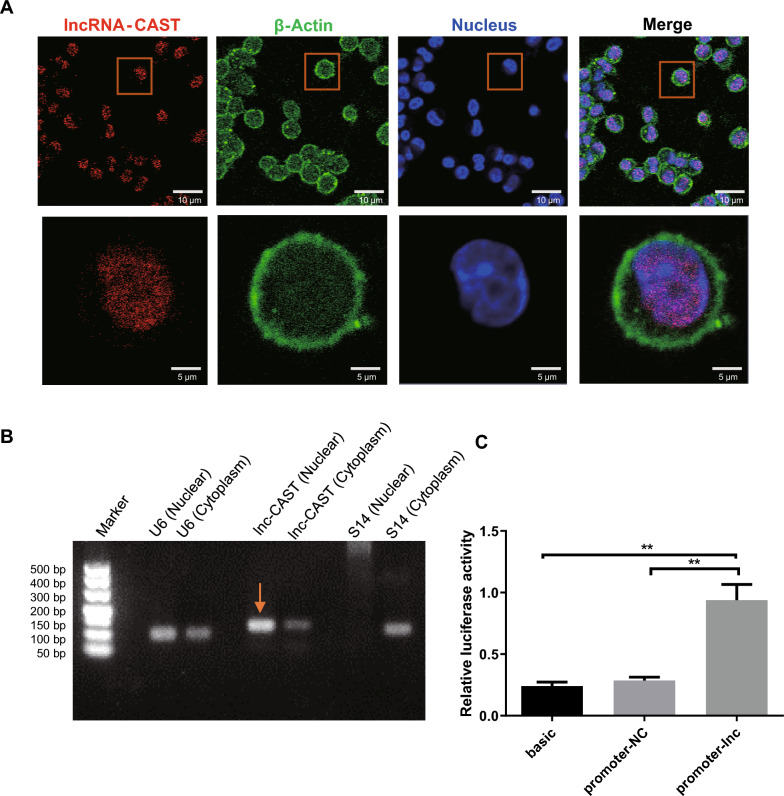
**Subcellular localization of promoter-associated lnc-CAST in PAM cells.** **A** Confocal microscopic images of RNA FISH assay of lnc-CAST showed that lnc-CAST colocalizes with the nucleus of PAM cells from SPF pigs. Lower images were cropped from the squares in the upper images (Scale bars: 10 μm in upper and 5 μm in lower). More than 30 cells were examined and had similar results. Orange block diagrams mark the nucleus/lnc-CAST colocalization. **B** Gel electrophoresis of lnc-CAST extracted from nucleus and cytoplasm of PAM cells. As controls, more S14 expressed in cytoplasm and more U6 expressed in nucleus, respectively. **C** Cell lysates of HEK-293T cells were harvested for dual-luciferase assays, and the relative luciferase activity was quantified. The data represent the means for triplicate samples from one independent experiment. The experiments were repeated twice. *P < 0.05; **P < 0.01 (*t* test).

To elucidate the potential mechanisms of lnc-CAST, we examined its subcellular distribution in PAMs. We designed an RNA probe specifically for lnc-CAST, and its specificity was validated by Northern blot (Additional file 5). We then conducted FISH analysis using this probe in conjunction with an anti-β-actin antibody, facilitated by confocal microscopy. The results indicated that lnc-CAST is primarily located in the nucleus of PAMs (Figure 7A). To further verify the subcellular distribution of lnc-CAST, we separated and collected the cytoplasmic and nuclear fractions of PAMs, and carried out gel electrophoresis on the extracted RNA. In line with our previous findings, the bulk of lnc-CAST was found to be concentrated in the nuclear fraction (refer to Figure 7B). These observations lead us to hypothesize that lnc-CAST might physically interact with chromatin, thereby significantly enhancing the transcription of CXCL8.

To ascertain whether lnc-CAST has a direct impact on the expression of the *Cxcl8* gene, we engineered a fragment of the *Cxcl8* promoter region into pGL3-Basic plasmid, which was subsequently transfected into 293T cells. The double luciferase activity assay results revealed that the overexpression of lnc-CAST resulted in an elevated luciferase activity driven by the *Cxcl8* promoter, in comparison to the negative control (Figure 7C). Taken together, these observations suggest that lnc-CAST, a nuclear lncRNA with *cis*-acting properties, binds to the *Cxcl8* promoter, thereby facilitating the expression of the *Cxcl8* gene.

### Lnc-CAST enhances CXCL8 transcriptional activity through H3K27ac

**Figure 8:**
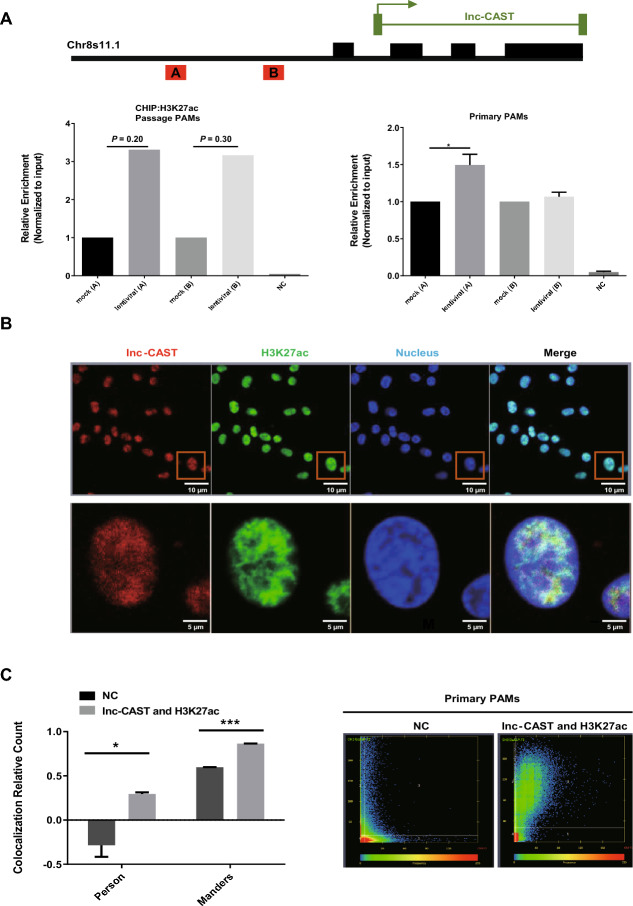
**Lnc-CAST directly recruits H3K27ac to CXCL8 promoter.** **A** ChIP-qPCR analysis of H3K27ac and control antibodies at the promoters of CXCL8 in immortalized (left) and primary (right) PAM cells transfected with lentiviral-lnc-CAST vector. **B**–**C** Confocal microscopic images of RNA FISH assay of lnc-CAST and immunofluorescence analysis of H3K27ac showed that H3K27ac colocalizes with lnc-CAST in PAM cells from SPF pigs. Lower images were cropped from the squares in the upper images (scalebars: 10 μm in upper and 5 μm in lower). More than 30 cells were examined and had similar results. Orange block diagrams mark the H3K27ac/lnc-CAST colocalization. Statistical significance (*t* test): *P < 0.05; **P < 0.01 ***P < 0.001. Error bars represent SEM from three independent experiments.

H3K27ac is widely recognized as a marker for active promoters and distal enhancers [32]. Thus, to investigate if lnc-CAST could enhance the H3K27ac modification at various regions of the *Cxcl8* gene promoter, we carried out ChIP-qPCR. The results indicated a significant enrichment of H3K27ac near the promoter region of the *Cxcl8* gene when lnc-CAST was overexpressed, compared to other regions or the control (Figure 8A). To delve deeper into the relationship between lnc-CAST and H3K27ac, we conducted a confocal microscopy-based FISH analysis using the lnc-CAST probe and an anti-H3K27ac antibody. As depicted in Figure 8B, a considerable amount of H3K27ac was found to colocalize with lnc-CAST in the nucleus of PAMs. Further colocalization analyses affirmed the specific association between lnc-CAST and H3K27ac (Figure 8C). Collectively, these findings suggest that lnc-CAST may play a role in facilitating the recruitment of H3K27ac to the promoter loci of the *Cxcl8* gene, thereby regulating CXCL8 transcription.

## Discussion

The innate immune system plays a crucial role in the initial defense against viral infections, with cytokines serving as key mediators in cell-to-cell communication, inflammatory response amplification, and immune regulation [33]. Published data show that the vast majority of cytokines are produced during PRRSV infection in vivo and in vitro [13, 34]. Among them, some proinflammatory cytokines including IL-1α, TNF-α, and IL-8, are of greatest importance in interfering with the different steps of immunity in persistent infection [35, 36]. LncRNAs have emerged as critical regulators of gene expression, particularly in immune regulation [13]. In this study, we introduced a novel lncRNA named as lnc-CAST and identified the function of lnc-CAST in the immune response of PAMs. A previous study observed that the expression levels of numerous lncRNAs were perturbed during PRRSV infection [26]. Among them, lnc-CAST is significantly up-regulated upon PRRSV infection, indicating its potential involvement in the inflammatory response.

LncRNAs were first discovered to have gene-specific regulatory roles in the early 1990s with the discovery of lncRNAs involved in epigenetic regulation, such as H19 [37] and Xist [38]. Since then, regulatory lncRNAs have been characterized in various species, participating in diverse processes such as inflammatory mediators, differentiation, and cell migration, revealing a new layer of regulation in eukaryotic cells [39]. This discovery was made possible by the advent of high-throughput sequencing. For instance, Imamura et al. demonstrated that upregulation of NEAT1 leads to sequestration of paraspeckle proteins, which causes activation of CXCL8 transcription and executes its repressor function upon HDV infection [40, 41]. Here, we obtained the full-length sequence of lnc-CAST and performed bioinformatic analysis, revealing its cis-acting nature to its putative target gene of *Cxcl8*. Furthermore, we determined that lnc-CAST was a potentially functional regulatory molecule that possessed tissue- and cell-specific expression. To establish an in vivo correlation, we evaluated the RNA expression of lnc-CAST and CXCL8 in PRRSV-infected lung samples by RT-qPCR, and protein expression of CXCL8 by ELISA. Hence, we demonstrated that lnc-CAST could be upregulated during PRRSV infection and subsequently induces CXCL8 production in vivo. Additionally, consistent with the in vivo results, upon diverse inflammation responses, PRRSV, LPS, LTA, and Poly (I:C) induced higher level of lnc-CAST and CXCL8 mRNA expression in PAMs than from the control. Therefore, we hypothesized and demonstrated that lnc-CAST binds to promote CXCL8 expression, and can directly regulate the RNA and protein expression of CXCL8.

In humans, the chemokine CXCL8 is a powerful inducer of directional cell motility, primarily during inflammation. CXCL8 stimulation attracts neutrophils to inflammation site [42], and induces migration, invasion, and proliferation in different cell types expressing CXCL8 receptors CXCR1 and CXCR2 [43, 44]. Based on our data, we demonstrated that lnc-CAST acts as a positive regulator of CXCL8 release in the culture supernatant of PAMs. Thus, we further examined the regulatory function of lnc-CAST-stimulated supernatant on various cell types, including neutrophils, IPEC, PT-K75 and PK-15 cells. Our results demonstrated that lnc-CAST-mediated supernatant could facilitate migration and wound repair of these target cells.

Next, we explored the mechanism underlying the effects of lnc-CAST on CXCL8. The regulatory potential of lncRNAs is closely correlated with their specific subcellular location within the cell including the nucleus, chromatin and cytosol [45]. Among them, a significant number of nuclear lncRNAs associate with chromatin and thus could be broadly classified as cheRNAs (chromatin-enriched RNAs) [46]. Some nuclear lncRNAs can influence chromatin architecture by interacting with chromatin modulating proteins and promoting their recruitment/association to chromatin, thereby controlling transcriptional activity [47–54]. For example, in hepatocarcinoma cells, lnc-TCF7 facilitates the transcription of TCF7 by recruiting the SWI/SNF complex to the TCF7 promoter [55]. Thus, we investigated the location of lnc-CAST in PAMs, and then found lnc-CAST are mainly colocalized in the nucleus. Therefore, we hypothesized whether lnc-CAST binds to the promoter of CXCL8 or histone modification to facilitate the expression. Recently, several interesting observations were discovered at the promoter region. Gene expression is strongly associated with histone modification, especially porcine H3K27ac [56]. H3K27ac is known to shape active promoters and enhancers by opening chromatin, thereby allowing the transcriptional machinery to assemble at the core promoter [57]. To establish a correlation between lnc-CAST and CXCL8, we evaluated the expression of lnc-CAST and the promoter of CXCL8 in 293T cell by dual luciferase report assay. Consistent with our primary hypothesis, the increased expression lnc-CAST induced significantly higher fluorescence signal of CXCL8 promoter. Additionally, we further explored the lnc-CAST-H3K27ac relationship by CHIP and colocalization in PAMs. Based on our data, we demonstrated that the lnc-CAST recruits the H3K27ac to facilitate the activation of CXCL8 promoter and the transcription of CXCL8. However, further animal and clinical studies are required to test this hypothesis. Similar to our findings, other lncRNA such as Lnc-MxA [58] and lnc-TCF7 [55] have also been reported to form or recruit a complex at its promoter to regulate target gene transcription.

In summary, our study uncovers a novel lncRNA, lnc-CAST, which is involved in immune responses and is upregulated with various stimuli in vitro and in vivo, including PRRSV infection. In vitro, lnc-CAST augments migration upon a wide variety of cell lines by upregulating the expression of CXCL8. This effect is mediated by the recruitment of H3K27ac to facilitate the activation of CXCL8 promoter, though the signaling pathway about lnc-CAST remains to be elucidated. In conclusion, we have uncovered a novel porcine lncRNA and a novel mechanism in which lnc-CAST recruits H3K27ac to facilitate the transcription of CXCL8 and cell migration.

### Supplementary Information

**Additional file 1.** **Primers used for PCR.**

**Additional file 2.** **Primers used for RT-qPCR.**

**Additional file 3.** **Primers used for plasmids.** This table provides the primers for plasmids (Dual-Luciferase and pLVX-IRES-ZsGreen).

**Additional file 4.** **Detection of full-length lnc-CAST transcript.** This figure provides the evidence of full-length lnc-CAST transcript. (A) Results of reverse transcription PCR of the lnc-CAST transcript. The vertical arrow shows the length of observed products in base pairs (bp). (B) Results of 5′- and 3′-RACE analysis is presented. The vertical arrows represent the exact 5′- and 3′-ends. (C) Comparative study of the full-length lnc-CAST transcript with Cxcl8 of sus scrofa gene (GenBank: AB 057440.1). (D) Comparative study on alignment of the full-length lnc-CAST transcript via UCSC genome browser.

**Additional file 5.** **lnc-CAST in Northern blot analysis.** An RNA probe specific for lnc-CAST was designed, and its specificity was confirmed by Northern blot analysis.

## Data Availability

The transcriptome data of porcine macrophages used for this study are available in the NCBI database under the Bioproject accession number PRJNA658105 (Biosamples SAMN15857517 to SAMN15857534). The supporting data for the findings of this study can be obtained from the corresponding author upon reasonable request.
