# Supplementary material for: Porcine cis-acting lnc-CAST positively regulates CXCL8 expression through histone H3K27ac
Source: Vet Res. 2024 May 7;55:56. doi: 10.1186/s13567-024-01296-9 (PMC11077775; doi:10.1186/s13567-024-01296-9)
Supplement: Supplementary file 1 — Additional file 1. Primers used for PCR. [file 13567_2024_1296_MOESM1_ESM.pdf]

**Primer**	**Sequence (5'-3')**
Lnc-CAST-F	CATGTAAGTAATTTCAAAAGTGAT
Lnc-CAST-R	ATAAGTGAATAAATTACACTTG
5’-RACE-GSP/R	AGCTCACTGTTGCTGCTAATTTCTTTGT
5’-RACE-NGSP/R	AACATCCTTTTGCCATTCAGGTCATGGT
3’-RACE-GSP/F	GAGCTTCTTGTACTTGTAATTGGGAGGT
3’-RACE-NGSP/F	CTCCCTCTCAATCTCAGAAGTGTTTAGA
porcine-HU6s-F	CGCTTCGGCAGCACATATAC
porcine-HU6s-R	TTCACGAATTTGCGTGTCATC
porcine-s14-F	ATCTGCAACCTATACCACAGC
porcine-s14-R	CCGACTAGGAACCATGAGATTG
porcine-HU6s-R	TTCACGAATTTGCGTGTCATC
porcine-s14-F	ATCTGCAACCTATACCACAGC
porcine-s14-R	CCGACTAGGAACCATGAGATTG
