# Supplementary material for: Porcine cis-acting lnc-CAST positively regulates CXCL8 expression through histone H3K27ac
Source: Vet Res. 2024 May 7;55:56. doi: 10.1186/s13567-024-01296-9 (PMC11077775; doi:10.1186/s13567-024-01296-9)
Supplement: Supplementary file 2 — Additional file 2. Primers used for RT-qPCR. [file 13567_2024_1296_MOESM2_ESM.pdf]

Primer	Sequence (5'-3')
Porcine-β-actin-F	CTTCCTGGGCATGGAGTCC
Porcine-β-actin-R	GGCGCGATGATCTTGATCTTC
CXCL8-F	CCGTGTCAACATGACTTCCAA
CXCL8-R	GCCTCACAGAGAGCTGCAGAA
CAST-F	GCAAAAGGATGTTGGAAACCC
CAST-R	TCTGCACCCACTTTTCCTTG
CHIP-CXCL8-F-A	GGTTGCGTAGTGTGGAATTTC
CHIP-CXCL8-R-A	GGGACTTGTGCTGGATGG
CHIP-CXCL8-F-B	GTAATTGTGCCATAAAAGAATAATCA
CHIP-CXCL8-R-B	GCATTGAGCTTCTTACCTGATCTTTC
