# Supplementary material for: Porcine cis-acting lnc-CAST positively regulates CXCL8 expression through histone H3K27ac
Source: Vet Res. 2024 May 7;55:56. doi: 10.1186/s13567-024-01296-9 (PMC11077775; doi:10.1186/s13567-024-01296-9)
Supplement: Supplementary file 3 — Additional file 3. Primers used for plasmids. This table provides the primers for plasmids (Dual-Luciferase and pLVX-IRES-ZsGreen). [file 13567_2024_1296_MOESM3_ESM.pdf]

Primer	Sequence (5'-3')
CAST-Xho1-F	CCGCTCGAGCATGTAAGTAATTTCAAAAGTGAT
CAST-Not1-R	ATAGTTTAGCGGCCGCATAAGTGAATAAATTACACTTG
pGL3-basic-F	TAAGCTTGGCATTCCGGTACTGTTG
pGL3-basic-R	CCGGGCTAGCACGCGTAAGAGCTCG
CXCL8-promoter-F (-493)	TACGCGTGCTAGCCCGGGTAATTGTGCCATAAAAGAATAAT
CXCL8- promoter-R (+59)	CCGGAATGCCAAGCTTACAGAGAGCTGCAGAAAGCAG
