# Supplementary figures and images for: Porcine cis-acting lnc-CAST positively regulates CXCL8 expression through histone H3K27ac
Source: Vet Res. 2024 May 7;55:56. doi: 10.1186/s13567-024-01296-9 (PMC11077775; doi:10.1186/s13567-024-01296-9)

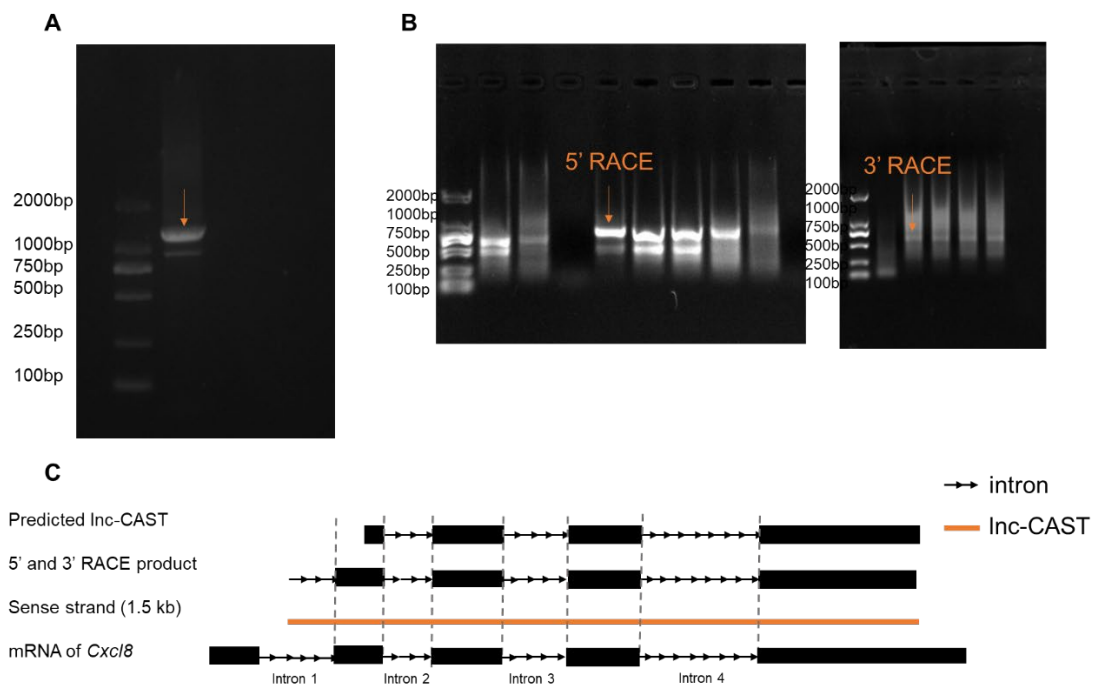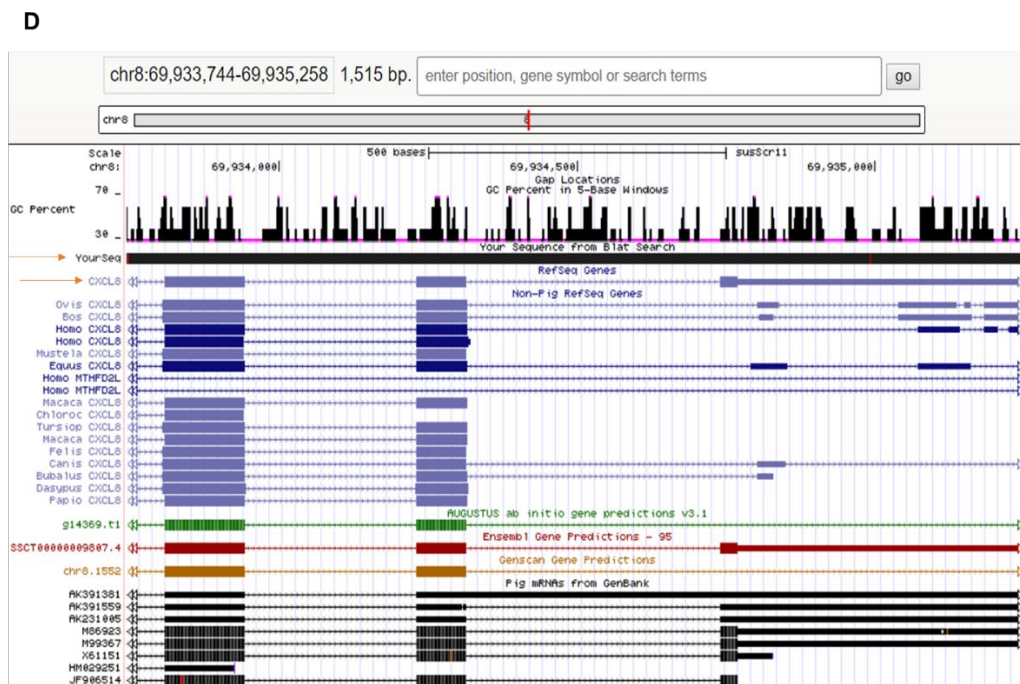

Supplement: Supplementary file 4 — Additional file 4. Detection of full-length lnc-CAST transcript. This figure provides the evidence of full-length lnc-CAST transcript. (A) Results of reverse transcription PCR of the lnc-CAST transcript. The vertical arrow shows the length of observed products in base pairs (bp). (B) Results of 5′- and 3′-RACE analysis is presented. The vertical arrows represent the exact 5′- and 3′-ends. (C) Comparative study of the full-length lnc-CAST transcript with Cxcl8 of sus scrofa gene (GenBank: AB 057440.1). (D) Comparative study on alignment of the full-length lnc-CAST transcript via UCSC genome browser. [file 13567_2024_1296_MOESM4_ESM.pdf]

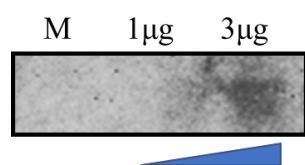

Supplement: Supplementary file 5 — Additional file 5. lnc-CAST in Northern blot analysis. An RNA probe specific for lnc-CAST was designed, and its specificity was confirmed by Northern blot analysis. [file 13567_2024_1296_MOESM5_ESM.pdf]
